# The impact of nevirapine- versus protease inhibitor-based regimens on virological markers of HIV-1 persistence during seemingly suppressive ART

**DOI:** 10.7448/IAS.17.4.19823

**Published:** 2014-11-02

**Authors:** Maja Kiselinova, Maria Anna, Eva Malatinkova, Karen Vervish, Apostolos Beloukas, Peter Messiaen, Pawel Bonczkowski, Wim Trypsteen, Steven Callens, Chris Verhofstede, Ward De Spiegelaere, Linos Vandekerckhove

**Affiliations:** 1 Internal Medicine and Infectious Diseases, Ghent University Hospital, Ghent, Belgium; 2 Clinical Infection, Microbiology and Immunology, University of Liverpool, Liverpool, UK; 3 Infectious Diseases and Immunity, Jessa Hospital, Hasselt, Belgium; 4 AIDS Reference Laboratory, Ghent University Hospital, Ghent, Belgium

## Abstract

**Introduction:**

The source and significance of residual plasma HIV-1 RNA detection during suppressive ART remain controversial. It has been proposed that nevirapine (NVP)-based regimens achieve a greater HIV-1 RNA suppression than regimens containing a protease inhibitor (PI). The aim of this study was to compare the effect of receiving NVP- vs PI-based ART on the virological markers of HIV persistence in peripheral blood.

**Material and Methods:**

The study population comprised 161 HIV-1 infected patients receiving either NVP-based (n=81) or PI-based (n=80) ART and showing a HIV-1 RNA load stably suppressed <40 copies/mL for median of 5.2 years (IQR 2.2–8.0). Residual viraemia was detected by real-time PCR with 50% and 95% detection thresholds of 1 and 3 HIV-1 RNA copies/mL, respectively. Cell-associated (CA) unspliced HIV-1 RNA, total HIV-1 DNA and 2 LTR circles were quantified in peripheral blood mononuclear cells (PBMCs) using droplet digital PCR. Groups were compared by standard non-parametric tests; factors associated with HIV-1 detection were analyzed by univariate regression analysis and generalized linear models (SPSS^®^ V22 and Rstudio).

**Results:**

Plasma HIV-1 RNA was detected in 37/81 (45.7%) and 47/80 (58.8%) subjects on NVP- and PI-based ART, with median (IQR) levels of 5 (3–6) and 5 (3–8) copies/mL, respectively. HIV-1 RNA detection was associated with shorter duration of suppressive ART regardless of treatment arm (p=0.007), and lower CD4 nadir (p=0.015). HIV-1 DNA levels were median 282 (120–484) and 213 (87–494) copies/106 PBMCs in the two groups respectively, and were lowest (<100 copies/106 PBMCs) in subjects with lower plasma HIV-1 RNA (p=0.049), CA unspliced HIV-1 RNA (p=0.0001), 2 LTR circles (p=0.005) and pre-ART HIV-1 RNA load (p=0.0001).

**Conclusions:**

In this comprehensive characterization of patients on long-term suppressive ART, we did not observe evidence for a greater suppressive activity of NVP-based over PI-based therapy on plasma and intracellular markers of virus persistence. Overall excellent correlation was observed between the markers, allowing the identification of a subset of treated patients with low HIV-1 expression as an important cohort for future HIV cure studies.

